# Accumulated lifetime violence load and interpersonal problems among suicidal women with emotionally unstable personality disorder

**DOI:** 10.3389/fpsyt.2025.1646158

**Published:** 2025-08-28

**Authors:** Fredrik Gustafsson, Adrian Desai Boström, Alexander Wilczek, Matilda Naesström, Mia Rajalin, Marie Åsberg, Jussi Jokinen

**Affiliations:** ^1^ Department of Clinical Sciences/Psychiatry, Umeå University, Umeå, Sweden; ^2^ Department of Clinical Neuroscience, Karolinska Institutet, Stockholm, Sweden; ^3^ Department of Clinical Sciences, Karolinska Institutet, Stockholm, Sweden

**Keywords:** emotionally unstable personality disorder, women, suicide attempt, suicide, victims of violence, perpetrators of violence, interpersonal problems, childhood trauma

## Abstract

**Background:**

Patients with emotionally unstable personality disorder (EUPD) report increased use and exposure to interpersonal violence and experience significant interpersonal difficulties. The relationship between the type of self-reported interpersonal problems and interpersonal violence among individuals with EUPD remains unclear. This study investigates the association between interpersonal problems and the cumulative lifetime violence burden among suicidal women with EUPD.

**Methods:**

The study included 103 women diagnosed with EUPD who had attempted suicide at least twice. The inventory of Interpersonal problems (IIP) and the Karolinska Interpersonal Violence Scale (KIVS) were used to assess interpersonal problems and lifetime violence load. The eight IIP subscales were grouped into two factors: Factor 1 (Domineering; Vindictive; Cold; Social avoidant) and Factor 2 (Nonassertive; Exploitable; Overly nurturant, and Intrusive).

**Results:**

The mean T-scores for IIP subscales ranged from 56 to 63, while the KIVS total score varied from 0 to 19, with a mean of 7.63. Cumulative lifetime violence burden was significantly associated with IIP Factor 1 (Spearman’s rho=0.26, p=0.0081), but not with IIP Factor 2 (r=0.014, p=0.89). Linear regression analysis revealed that Factor 1 (t=2.99, *p*=0.0039) and global assessment of functioning (GAF) (t=-2.26, *p*=0.027) significantly predicted accumulated lifetime interpersonal violence. Further *post-hoc* analysis showed that individuals with higher violence burdens exhibited significantly greater interpersonal problems in the three domains of Factor 1 - Domineering/Controlling, Vindictive/Self-centered, and Cold/Distant.

**Conclusions:**

Specific Interpersonal problems and low functioning seem to be associated with cumulative lifetime violence burden among suicidal women with EUPD. These interpersonal traits may serve as key targets for treatment and prevention.

## Highlights

- Women with emotionally unstable personality disorder report high levels of interpersonal violence and interpersonal problems.
- High violence burden is associated with being controlling, self-centered and distant in interpersonal relationships.
- Low global functioning is associated with cumulative lifetime violence burden among suicidal women with emotionally unstable personality disorder.

## 1 Introduction

Emotionally unstable personality disorder (EUPD), also known as Borderline Personality Disorder, one of cluster B disorders, is characterized by nine symptoms/criteria such as emotional dysregulation, chronic feelings of emptiness, self-destructive impulsivity and instability in interpersonal relationships (1). The disorder is associated with increased mortality, including a nearly five-fold increase in all-cause mortality and a 50-fold increase in suicide risk (2, 3). Interpersonal problems are central to EUPD pathology and play a crucial role in treatment outcomes (4). Difficulties in interpersonal relationships have also proved to be a trigger for suicidal behavior, and even important in the familial transmission of suicidal behavior (5, 6).

Exposure to violence during childhood and violent behavior in adulthood are risk factors for later completed suicide in suicide attempters (7) and women with EUPD report high life-time interpersonal violence exposure (8). People with EUPD are more prone to use violence according to a recent systematic and meta-regression analysis (Odds ratio 2.6) (9). EUPD is characterized by a wide variety of interpersonal problems related to symptom severity and therapeutic alliance (10). Whether interpersonal violence is associated with specific types of interpersonal problems among individuals with EUPD is to our knowledge not known. This study investigates the association between interpersonal problems and cumulative lifetime interpersonal violence burden among women with EUPD and a history of repeated suicidal behavior.

## 2 Materials and methods

### 2.1 Participants

This study utilized data from 106 women diagnosed with EUPD who participated in the “Stockholm County Council and Karolinska Institute Psychotherapy Project for Suicide-Prone Women” (SKIP). Participants were recruited between 1999 and 2004 as part of a randomized controlled trial comparing dialectic behavioral therapy, psychodynamic therapy and treatment as usual. Inclusion criteria required a verified EUPD diagnosis and a history of at least two potentially lethal suicide attempts, with one occurring within six months before referral. Participants were excluded if they had a current life-threatening eating disorder, current psychotic disorder or major depressive illness with melancholic features, evidence of dementia or other irreversible organic brain syndrome or a current diagnosis of substance dependence. Details on the EUPD cohort have been previously published (8, 11). The original study protocol was approved by the Committee for Ethical Research at Karolinska Institutet (Dnrs: 95–283; 2021-06929-01). Patients were referred from all the psychiatric clinics in Stockholm County Council (encompassing care of 1.8 million inhabitants). A total of 162 women with EUPD were invited to take part in the SKIP project. Of these individuals, 14 (8.7%) declined to join the study, 41 (25.3%) were excluded due to not fulfilling inclusion criteria or to fulfilled exclusion criteria. One completed suicide before joining the study. All participants were checked in Swedish cause of death registry until 2011, and eight had died by suicide.

### 2.2 Assessments

The participants were interviewed by trained clinicians using a range of semi-structured psychiatric diagnostic and assessment tools, the SCID I research version interview to establish the DSM–IV diagnoses (12), and DIP–I to establish Axis II diagnoses (13). To measure the participants overall psychosocial impairment the Global Assessment of Functioning (GAF) was used (14). Non-violent suicide attempt method included tablet intoxications while all other methods were defined as violent (hanging, drowning, jumping from a height for example). The two questionnaires used to examine interpersonal problems and interpersonal violence are presented below.

#### 2.2.1 Inventory of interpersonal problems

The Inventory of Interpersonal Problems (IIP), a 64-item questionnaire measuring interpersonal problems has been extensively validated and has shown strong reliability (Cronbach’s alpha of 0.78) (15). Validity of the IIP has been confirmed in Sweden as well (16).

The inventory comprises eight subscales, each representing distinct interpersonal difficulties, with updated names provided in parentheses: Domineering (Domineering/Controlling), Vindictive (Vindictive/Self-centered), Cold (Cold/Distant), Social avoidant (Social inhibited), Non-assertive (Non-assertive), Exploitable (Overly Accommodating), Overly nurturant (Self-sacrificing), and Intrusive (Intrusive/Needy). Respondents indicate the extent to which they find it challenging to handle distressing interpersonal situations using statements like “it is hard for me to…” and “these are things I do too much or too often.” Responses are recorded on a Likert scale ranging from 0 (not at all) to 4 (extremely).

For instance, the Domineering scale assesses difficulties in maintaining control and exhibiting aggressive behaviors, such as “I try to change people too much.” High scores on the Vindictive scale indicate struggles with harboring vindictive thoughts and managing frustration and anger, as evidenced by statements like “it’s hard for me to put someone else’s needs before my own.” Similarly, a high score on the Cold scale suggests challenges in forming connections with others, with items such as “I keep other people at a distance too much.” The Social avoidant scale reflects a tendency to shy away from social interaction and express feelings, as seen in statements like “it’s hard for me to show my feelings.”

Scores on the Non-assertive scale reveal issues related to low self-confidence and difficulty in expressing needs assertively, exemplified by statements like “it’s hard for me to be firm when I need to be.” High scores on the Exploitable scale indicate a propensity to prioritize others’ needs over one’s own and difficulties in expressing anger, as demonstrated by statements like “it is hard for me to let other people know when I’m angry.”

In contrast, the Overly nurturant scale highlights struggle with setting boundaries and prioritizing others’ needs excessively, as shown in statements like “I put other people’s needs before my own too much.” Lastly, the Intrusive scale assesses challenges in respecting others’ boundaries, as evidenced by statements like “I tell personal things to other people too much.”

The total score, transformed into a normative T-score, reflects an individual’s overall level of interpersonal issues compared to the general population. Scores on the eight scales delineate specific problematic areas and types of interpersonal problems. Additionally, an ipsative T-score can offer insights into an individual’s personal level of interpersonal difficulties. These scores can be utilized to assess a patient’s interpersonal issues, compare different groups, or measure changes in interpersonal problems pre- and post-treatment.

The eight IIP subscales are known to intercorrelate and to reduce the risk of multicollinearity, the scales could be grouped into two factors as follows: Factor 1 (Domineering; Vindictive; Cold; Social avoidant) and Factor 2 (Nonassertive; Exploitable; Overly nurturant, and Intrusive) using normative T-scores (15, 17).

#### 2.2.2 Karolinska interpersonal violence scale

The Karolinska Interpersonal Violence Scale (KIVS): a structured interview measuring both exposure and expressed violent behavior in childhood (defined as 6–14 years of age) and during adult life (defined as age 15 or older). The ratings are based on a structured interview with concrete examples of interpersonal violence performed and assessed by trained clinicians. It was first published in 2010 (7) and validated using the Buss-Durkee Hostility Inventory (BDHI) (18), “Urge to act out hostility” subscales from the Hostility Questionnaire (HDHQ) (19) and the Early Experience Questionnaire (EEQ) (20). The complete scale is presented in **Appendix 1**.

In brief, the first step of the KIVS explores the subjects experience of using violence as a child and as an adult and is rated 0–5 where a high number indicate a history of violent behavior. The total maximal score is 20. For example, the score “3” for used violence as a child were stated as “Often started fights. Hit comrade who had been bullied. Continued hitting when other had surrendered” and the highest score “5” was defined as “Caused serious physical injury. Violent toward adult(s). Violent behavior that led to intervention by social welfare authorities”. The second part on the other hand focuses on the subject as a victim of violence, again divided by experience as a child and as an adult. The “5” score for being a victim of violence during adulthood were stated as “Repeatedly raped. Repeatedly battered. Severely battered, resulting in serious bodily harm”.

### 2.3 Statistical methods

Shapiro Wilks test evaluated data distribution. Correlational analyses were used to determine associations between the total score of interpersonal violence (KIVS total score) and the two IIP factors, age at first suicide attempt, GAF score as well as the number of EUPD criteria, as continuous variables. Pearson’s r was applied for parametric correlation analyses and Spearman´s rho was applied for non-parametric correlation analyses. Depending on whether the data was normally distributed or not we used Student’s t-test or Kruskal-Wallis’ test to assess group differences (suicide attempters with and without violent suicide attempt method, respectively) in continuous variables.

From the results of the bivariate analyses of the two IIP factors, a multiple linear regression analysis was conducted to determine whether accumulated interpersonal violence was associated with specific domain of interpersonal problems adjusted for the number of EUPD criteria and the GAF score. The Durbin—Watson test statistic expressed no correlation in adjacent residuals. All statistical tests were two-tailed.

Statistical significance was set at *p <*0.05. We used the Statistical Package JMP 9.0.3 software, SAS Institute Inc., Cary, NC, USA.

## 3 Results

### 3.1 Participants characteristics

The mean age was 30 years (range 19–50 years, SD=7.8). 85% (n=90)of the patients had a mood disorder diagnosis and 83% (n=88) met criteria for anxiety disorders. 57% (n=60) of the patients had post-traumatic stress disorder (PTSD) and 26% (n=27) had a comorbid eating disorder. 54% (n=57) of the patients fulfilled the criteria for substance use disorder past or present (all substances, alcohol included). Fifty women had an additional personality disorder and 24% (25%) of women had three or more personality disorders. The mean age at first suicide attempt was 20 years (range 5-46, SD=7.6). Eleven percent of women with EUPD reported having used a violent suicide attempt method. The mean T-scores for IIP subscales ranged between 56–63 and are shown in **Table 1**. More on participants characteristics can be found in **Table 2**. The mean KIVS total score was 7.63 (range 0-19, SD=4). Mean number of EUPD criteria was 6.2 and 36 percent fulfilled 7 or more EUPD criteria. Mean score of the Global Assessment of Functioning (GAF) was 49.

**Table 1:** IIP-ratings in suicidal women with EUPD (n=103), T-score mean, standard deviation (SD), range.

IIP Subscales	T score Mean (SD)	Range
Domineering	58 (14)	39-98
Vindictive	59 (17)	39-88
Cold	60 (14)	39-96
Social avoidant	63 (15)	37-99
Non-assertive	60 (13)	36-86
Exploitable	59 (14)	33-90
Overly nurturant	61 (12)	34-90
Intrusive	59 (12)	37-89

**Table 2:** Characteristics of subjects.

Characteristics of subjects	
N	106
Age (years), mean (SD)	30 (7.8)
KIVS total score, mean (SD)	7.63 (4)
Use of violent suicide attempt method n (%)	11 (10.6)
Number of DSM-V EUPD criteria, mean (SD)	6.2 (1.2)
≥7 fulfilled EUPD criteria, n (%)	38 (36)
Global Assessment of Functioning (GAF), mean (SD)	49 (12)
Later confirmed death by suicide, n (%)	8 (8)
Comorbid substance use diagnosis, past or present n (%)	56 (54)

### 3.2 Correlations and regression analysis

**Figure 1:**
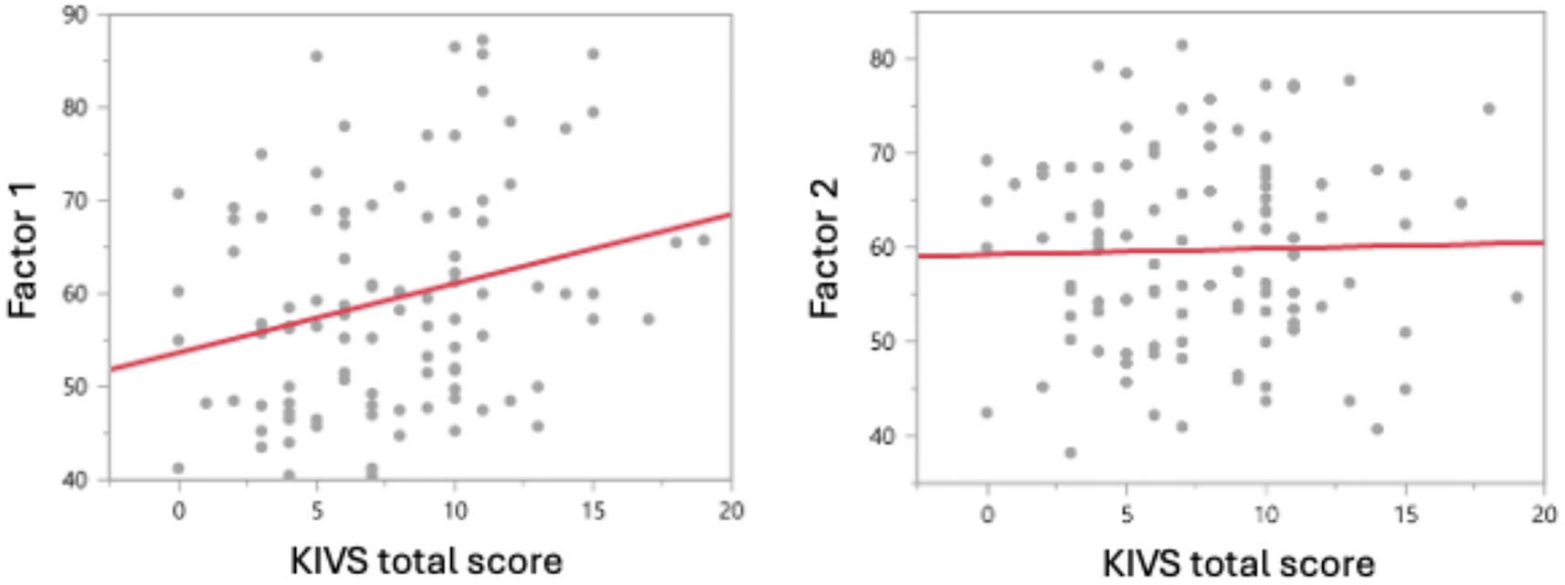
Bivariate correlation analysis of the Cumulative lifetime burden of violence, as measured by the total KIVS score, is significantly positively associated with Factor 1 (Spearman’s rho=0.26, p=0.0081), but not with Factor 2 (r=0.014, p=0.89).

The cumulative lifetime interpersonal violence was significantly associated with IIP Factor 1 (r=0.26, *p*=0.0071), but not with Factor 2 (r=0.014, p=0.89), **Figure 1**. Higher number of EUPD criteria was significantly associated both with both Factor 1 (r=0.38, *p*<0.0001) and cumulative lifetime interpersonal violence (r=0.26, *p*=0.0075). The correlation between Factor 1 and Factor 2 was significant (r=0.28, p=0.0049). GAF ratings showed significant negative correlation with the cumulative lifetime interpersonal violence (r= -0.25, *p*=0.025) Patients who had used a violent suicide attempt method (e.g. hanging, strangulation/suffocation, or firearms use) or patients with substance use disorder (past or present) did not have significantly different ratings in Factors 1 or 2 or KIVS total score or number of EUPD criteria. **Table 3** shows correlations between the KIVS total score, Interpersonal problems grouped into Factor 1 and Factor 2, number of EUPD criteria, age at onset of suicidal behavior and GAF.

**Table 3:** Correlations (Spearman’s p) between lifetime accumulated violence load and interpersonal problems grouped into Factor 1 vs Factor 2, age at first suicide attempt (SA) and number of EUPD criteria.

	Factor 1 IIP	Factor 2 IIP	Age at first SA	Number of EUPD criteria	GAF
KIVS total score	0.26*	0.014	-0.035	0.26*	-0.25*

p, p-value; IIP, Inventory of Interpersonal Problems; KIVS, Karolinska Interpersonal Violence Scale; GAF, global *p < 0.05.

A multiple linear regression analysis based on the results in correlation analyses was conducted with Factor 1, Factor 2, GAF and number of EUPD criteria as predictors of accumulated lifetime interpersonal violence, entered simultaneously as predictors. The regression model was significant, adjusted RSq=0.15, df=4, p=0.004. Factor 1 and GAF were significant predictors of accumulated lifetime interpersonal violence in the regression model, **Table 4**.

**Table 4:** Multiple linear regression analysis based on the results in **Table 3**.

	KIVS total score, bivariate correlations	KIVS total score, regression analysis t ratio, p-value
IIP Factor 1	0.26*	t=2.99, p=0.0039*
IIP Factor 2	0.014	t=-0.77, p=0.44
GAF	-0.25*	t=-2.26, p=0.027*
Number of EUPD criteria	0.26*	t=1.78, p=0.079

*p < 0.05.

Further posthoc subscale analysis revealed that suicidal women with EUPD with a high burden of violence exhibited significantly greater interpersonal problems in the domains of Domineering/Controlling, Vindictive/Self-centered, and Cold/Distant, but not in the remaining five domains of interpersonal problems, **Table 5**.

**Table 5:** Subscale analysis of the Interpersonal problems in Factor 1 in correlation to lifetime accumulated violence load.

	Domineering	Vindictive	Cold	Social avoidant
KIVS total score	0.29**	0.26**	0.22*	0.12

*p < 0.05, **p < 0.01.

## 4 Discussion

In this study we assessed the association between Interpersonal problems and lifetime accumulated violence load among suicidal women with EUPD. The participants rated high levels of interpersonal problems, with a mean t-scores between 58 and 63 which is higher compared to a similar study that investigated exposure to early life adversity and interpersonal functioning in attempted suicide among both men and women where the mean t-score for the eight interpersonal problems ranged between 49-61 (5). The level of interpersonal violence was also high, which is in line with previous research that concluded that combined violence (self- and other directed violence) is more common among EUPD patients compared to a normal population with a prevalence of 70.7% (21). Patients were severely ill with high level of psychiatric comorbidity as well as a high number of EUPD criteria and low global functioning.

The main finding was that Factor 1 and GAF were significant predictors of accumulated lifetime interpersonal violence. Within Factor 1, three specific domains of interpersonal problems - Domineering, Vindictive and Cold – were significantly associated with the lifetime burden of interpersonal violence in suicidal women with EUPD. The remaining interpersonal problems mainly clustered in factor 2 – Social avoidant, Non-assertive, Exploitable, Overly nurturant and Intrusive - showed no significant correlations with interpersonal violence load. This indicates that suicidal women with EUPD and a history of violence both as a victim and a perpetrator have severe specific interpersonal difficulties. They show features as difficulties to let go of control and a proneness to aggression towards others (Domineering), emotional dysregulation reflecting problems with anger, frustration and vindictive thoughts (Vindictive) and problems in connecting to others and having problems to get along with other people (Cold).

We adjusted the analysis for the number of EUPD criteria since the number of criteria was significantly positively associated with both the total interpersonal violence load and Factor 1 in bivariate analysis. In the regression analysis, the more severe EUPD, e.g. higher number of met criteria for EUPD, showed a trend to be significantly associated with IIP Factor 1. This is partly in line with the results from another study which found that childhood trauma severity positively predicted EUPD symptom severity (22).

Interestingly, neither accumulated lifetime burden of interpersonal violence or interpersonal problems were significantly associated with earlier onset of suicide attempts or use of violent suicide attempt method in this cohort of women with EUPD. Earlier study on suicide attempters found higher levels of interpersonal violence among patients who had used a violent suicide attempt method (7). Violent suicide attempt methods are more common among men and our finding in this study may reflect the fact that all participants were women. Interestingly an earlier study reported significant correlations between specific interpersonal problems like being more intrusive (clustered in Factor 2) and family history of suicide among patients with a major depressive disorder and a recent suicide attempt (6) indicating that proneness to certain type of interpersonal problems can be related to certain phenotypes of suicidal behaviors maybe in a gender specific manner. In another study specific interpersonal problems in patients with EUPD seem to affect aggressive behaviors (23).

With all this in mind, different interpersonal problems, and lifetime violence burden are part of very complex intermediate phenotypes underlying EUPD and suicidal behavior. Whether the development of specific interpersonal problems is a consequence of accumulated interpersonal violence burden or vice versa cannot be concluded from this study. However, knowledge about these associations could be important when developing treatment strategies. Future research discovering neural correlates of aggression in personality disorders could also benefit from our results, where e.g. our finding considering low global functioning and its association to interpersonal violence and EUPD symptom severity seems to be a highly relevant area in neuroimaging research aiming to examine personality disorder traits relevant to aggressive behavior (24).

Due to the available data set males were not represented in this study. This could be seen both as a strength and a limitation. The strength is by reducing potential gender confounders and the limitation is that no conclusion or treatment direction can be stated for male individuals with EUPD which may act as a knowledge gap for future research.

Since the study design is cross-sectional, it does not allow for establishing causal relationships between cumulative violence and interpersonal problems or the direction of these relationships. The Karolinska Interpersonal Violence Scale does not assess different types of violence (physical, sexual, emotional) or their context (family, intimate partner, institutional) separately, which can be seen as a limitation since a more detailed information could be clinically relevant.

The data collection was performed between 1999 and 2004, more than 20 years ago. This means that the EUPD diagnoses were set according to the DSM-IV manual, which in present clinical practice is replaced by DSM-5, which could affect the generalizability of the results to current clinical settings and social context. However, all diagnoses were established by using DSM criteria based structured interviews (research versions) by trained psychiatrists and psychologists. One could also argue that the patients fulfill the criteria for the alternative model of personality disorders (AMPD) (1), given the fact that they also showed low global functioning which is highlighted in criterion A where interpersonal functioning is highly relevant. Our results on certain interpersonal problems related to accumulated interpersonal violence burden add to the literature on the interpersonal functioning in women with EUPD.

## 5 Conclusion

Specific Interpersonal problems seem to be associated with cumulative lifetime violence burden among suicidal women with EUPD. These interpersonal traits may serve as key targets for treatment and suicide prevention.

## Data Availability

The original contributions presented in the study are included in the article/**Supplementary Material**. Further inquiries can be directed to the corresponding author.
