# Supplementary material for: Accumulated lifetime violence load and interpersonal problems among suicidal women with emotionally unstable personality disorder
Source: Front Psychiatry. 2025 Aug 28;16:1646158. doi: 10.3389/fpsyt.2025.1646158 (PMC12423032; doi:10.3389/fpsyt.2025.1646158)
Supplement: Supplementary file 1 [file DataSheet1.pdf]

Table 1. The Karolinska Interpersonal Violence Scale[a]

The steps of this scale are defined by short statements about violent behavior. On the basis of an interview with the subject, use the highest score where one or more of the statements apply.

A. Used violence.

As a child (6–14 years)

0 No violence.
1 Occasional fights, but no cause for alarm among grown-ups in school or in the family.
2 Fighter. Been in fights a lot.
3 Often started fights. Hit a comrade who had been bullied. Continued hitting when the other had surrendered.
4 Initiated bullying. Often hit other children, with fist or object.
5 Caused serious physical injury. Violent toward adult(s). Violent behavior that led to intervention by social welfare authorities.

As an adult (15 years or older)

0 No violence.
1 Slapped or spanked children on occasion. Shoved or shook partner or another adult.
2 Occasionally smacked partner or child. Fought when drunk.
3 Assaulted partner drunk or sober. Repeated corporal punishment of child. Frequent fighting when drunk. Hit someone when sober.
4 Instance of violent sexual abuse. Repeated battering/physical abuse of child or partner. Assaulted/attacked other persons frequently, drunk or sober.
5 Killed or caused severe bodily harm. Repeated instances of violent sexual abuse. Convicted of crime of violence.

B. Victim of violence.

Childhood (6–14 years)

0 No violence.
1 Occasional slaps. Fights in school, of no great significance.
2 Bullied occasionally for short period(s). Occasionally exposed to corporal punishment.
3 Often bullied. Frequently exposed to corporal punishment. Beaten by drunken parent.
4 Bullied throughout childhood. Battered/beaten up by schoolmates. Regularly beaten by parent or another adult. Beaten with objects. Sexually abused.
5 Repeated exposure to violence at home or in school that resulted at least once in serious bodily harm. Repeated sexual abuse, or sexual abuse that resulted in bodily harm.

Adulthood (15 years or older)

0 No violence.
1 Threatened or subjected to a low level of violence on at least one occasion.
2 Beaten by partner on occasion. Victim of purse snatching. Threatened with object.
3 Threatened with a weapon. Robbed. Beaten by someone other than partner. Frequently beaten by partner.
4 Raped. Battered.
5 Repeatedly raped. Repeatedly battered. Severely battered, resulting in serious bodily harm.

[a]© Copyright 2010, Jussi Jokinen, MD, PhD. The Swedish version of the Karolinska Interpersonal Violence Scale (KIVS) was translated into English by an authorized bilingual translator; the English version of the KIVS was then back-translated into Swedish and the equivalence was checked by the original authors. Copies can be obtained from the author.
